# The prediabetes conundrum: striking the balance between risk and resources

**DOI:** 10.1007/s00125-023-05890-y

**Published:** 2023-03-10

**Authors:** Martin B. Blond, Kristine Færch, Christian Herder, Dan Ziegler, Coen D. A. Stehouwer

**Affiliations:** 1 Clinical Prevention Research, Steno Diabetes Center Copenhagen, Herlev, Denmark; 2 Department of Biomedical Sciences, University of Copenhagen, Copenhagen, Denmark; 3 Institute for Clinical Diabetology, German Diabetes Center, Leibniz Center for Diabetes Research at Heinrich Heine University Düsseldorf, Düsseldorf, Germany; 4 German Center for Diabetes Research (DZD), Partner Düsseldorf, München-Neuherberg, Germany; 5 Department of Endocrinology and Diabetology, Medical Faculty and University Hospital Düsseldorf, Heinrich Heine University Düsseldorf, Düsseldorf, Germany; 6 CARIM School for Cardiovascular Diseases, Maastricht University, Maastricht, the Netherlands; 7 Department of Internal Medicine, Maastricht University Medical Centre+, Maastricht, the Netherlands

**Keywords:** Intermediate hyperglycaemia, Prediabetes, Risk, Stratified medicine

## Abstract

The current definition of prediabetes is controversial and subject to continuous debate. Nonetheless, prediabetes is a risk factor for type 2 diabetes, is highly prevalent and is associated with diabetic complications and mortality. Thereby, it has the potential to become a huge strain on healthcare systems in the future, necessitating action from legislators and healthcare providers. But how do we best reduce its associated burden on health? As a compromise between differing opinions in the literature and among the authors of this article, we suggest stratifying individuals with prediabetes according to estimated risk and only offering individual-level preventive interventions to those at high risk. At the same time, we argue to identify those with prediabetes and already established diabetes-related complications and treat them as we would treat individuals with established type 2 diabetes.

**Graphical abstract:**

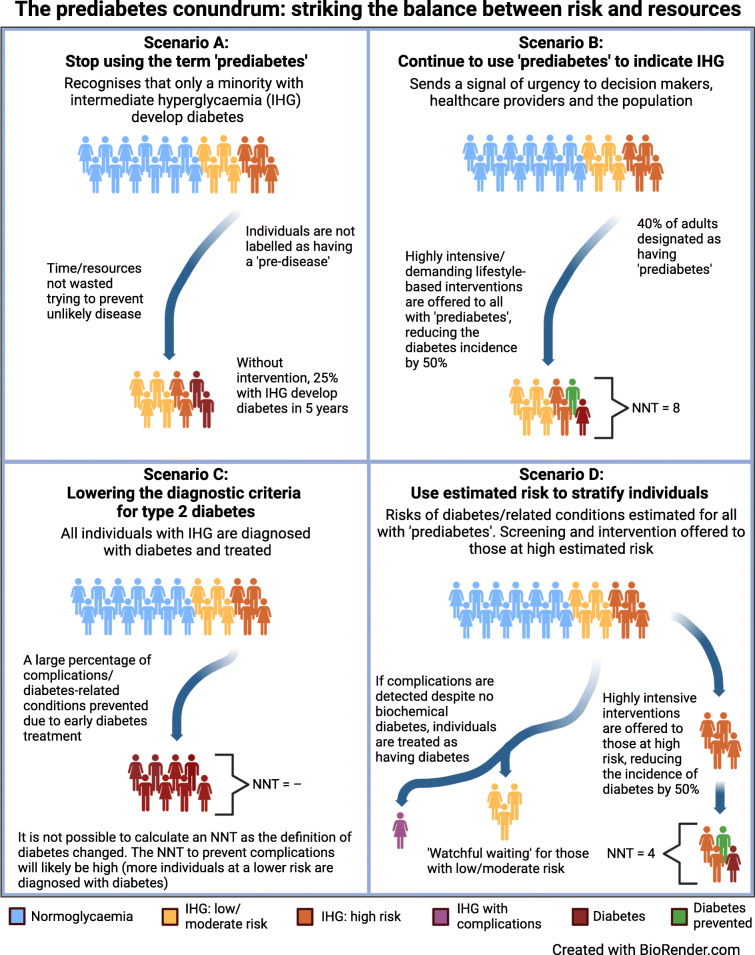

**Supplementary Information:**

The online version of this article (10.1007/s00125-023-05890-y) contains peer-reviewed but unedited supplementary material.

## Introduction

More than 20 years ago the ADA replaced the terms ‘impaired glucose tolerance’ (IGT) and ‘impaired fasting glucose’ (IFG) with ‘prediabetes’ in their standards of care [1], a controversial move that has since sparked heated debate [2–4]. Proponents of the change argue that the term is useful to realise the individual-level preventive potential, whereas opponents argue that labelling all individuals with intermediate hyperglycaemia as having a pre-disease medicalises a large proportion of the population. The discord extends to how to define prediabetes/intermediate hyperglycaemia and how to approach the individuals identified with prediabetes (Table 1). Despite the controversy, the term prediabetes has found its use with (some) researchers, patient organisations, medical societies, healthcare providers and governmental organisations.

**Table 1:** Examples of diagnostic criteria for prediabetes/intermediate hyperglycaemia and recommended actions from different organisations

Diagnostic criteria/recommendations by different organisations	ADA [45]	American Association of Clinical Endocrinologists [46]	WHO [15]	IDF [47]	International Expert Committee [18], ^a^
Term used	Prediabetes	Prediabetes	IFG/IGT	IFG/IGT	Subdiabetic hyperglycaemia
IFG	FPG: 5.6–7.0 mmol/l	FPG: 5.6–7.0 mmol/l	FPG: 6.1–7.0 mmol/l	FPG: 6.1–7.0 mmol/l	–
IGT	2hPG: 7.8–11.1 mmol/l	2hPG: 7.8–11.1 mmol/l	2hPG: 7.8–11.1 mmol/l	2hPG: 7.8–11.1 mmol/l	–
HbA_1c_ defined as high risk	HbA_1c_: 39–47 mmol/mol (5.7–6.4%)	HbA_1c_: 39–47 mmol/mol (5.7–6.4%)	–	–	HbA_1c_: 42–47 mmol/mol (6.0–6.4%)
Recommended action	Lifestyle behaviour change (weight loss, dietary counselling and physical activity) as well as metformin therapy for the prevention of T2D should be considered	Clinicians should actively treat people with prediabetes. The same targets are used for blood pressure, lipidaemia and weight as in T2D. Treatment includes lifestyle modification. Glucose-lowering medications can be used, and weight-loss medication should be considered in those with BMI ≥27 kg/m^2^	No specific recommendations targeting individuals with prediabetes. General recommendations regarding the importance of healthy body weight, being physically active, eating healthy and avoiding tobacco use	Comprehensive lifestyle programmes are cost-effective from a health system perspective. Local adaptations may be needed dependent on feasibility/access to healthcare services. Metformin can be considered a cost-effective strategy for T2D prevention, alongside comprehensive lifestyle programmes	When assessing risk, implementing prevention strategies or initiating a population-based prevention programme, other diabetes risk factors beyond glucose levels should be considered. Further analyses of cost–benefit should guide the selection of high-risk groups targeted for intervention within specific populations
Additional notes	Metformin is especially recommended for those aged 25–59 years with BMI ≥35 kg/m^2^, FPG ≥6.1 mmol/l, HbA_1c_ ≥42 mmol/mol (≥6.0%), and in women with prior gestational diabetes	The metabolic syndrome is regarded as a prediabetes equivalent. HbA_1c_ should be used only for screening for prediabetes. IFG or IGT should be confirmed with glucose testing	The cut-off points for IFG and IGT have not been updated since 2006	Follows WHO 2006 guidelines	The glucose-related terms describing subdiabetic hyperglycaemia will be phased out of use as clinical diagnostic entities as HbA_1c_ measurements replace glucose measurements for the diagnosis of diabetes

^a^The International Expert Committee consisted of members appointed by the ADA, the EASD and the IDF

2hPG, 2 h plasma glucose; T2D, type 2 diabetes

Why identify people with prediabetes? The prevalence of prediabetes is high and, on average, people with prediabetes are at increased risk of developing diabetes, diabetic complications and other related diseases, when compared with those with normoglycaemia [5, 6]. Consequently, the population-level health burden of prediabetes is substantial [7], underlining the necessity for early initiatives to prevent disease development. However, at the same time, there is large heterogeneity in individual risk among those with prediabetes. And herein lies the conundrum: how do we ensure early intervention in those with progressive disease, while also finding the balance between undertreatment and overtreatment? This balance is essential to avoid medicalisation and unnecessary stigma, to ensure a reasonable number needed-to-treat (NNT) in time- and resource-requiring prevention programmes, and to keep healthcare costs in check.

Opinions on how to approach prediabetes vary greatly. Some endorse the current definition [3], whereas others propose to rethink the approach to identify people at risk by including more markers of risk [8], while others want to abandon the prediabetes concept entirely [2]. It has even been suggested to lower the diagnostic threshold of diabetes to include the prediabetic range [9]. All these alternatives may have both positive and negative consequences (electronic supplementary material [ESM] Table 1).

With this article, we aim to give readers a brief overview of prediabetes and provide suggestions for potential routes going forward.

## Definition and history of prediabetes/intermediate hyperglycaemia

Type 2 diabetes is a multifactorial, multisystem metabolic disease. However, for historical and practical reasons we diagnose diabetes and prediabetes based only on blood glucose levels or HbA_1c_. Prediabetes is defined as the presence of intermediate hyperglycaemia in the form of at least one of the following: IGT, IFG or slightly elevated HbA_1c_ (Table 1). However, the overlap between individuals identified with prediabetes using the different criteria is relatively poor [10]. Moreover, despite the literature showing no clear threshold for the risk of later diabetes [11], specific but varying cut-off points are used to define prediabetes/intermediate hyperglycaemia (Table 1). IGT was introduced in 1979/1980 to cover the glucose range between diabetes and normal glucose tolerance [12]. The same dose of glucose is used for all individuals, and women generally have higher 2 h plasma glucose levels than men, at least in part due to differences in body size and distribution volume [13].

IFG was introduced in 1991 and was defined in such a way that the prevalence of IGT and IFG were similar in the study cohort of the Paris Prospective Study [14]. The lower cut-off point for IFG in the study was 6.1 mmol/l, the value still used by the WHO [15]. In 2003, this cut-off point was lowered to 5.6 mmol/l by the ADA [16], which increased the prevalence of IFG dramatically [17]. The WHO did not adopt the lower fasting plasma glucose criterion due to a lack of evidence of benefits in terms of reducing adverse outcomes [15].

In 2008, an expert committee with members from the ADA, the EASD and the IDF concluded that HbA_1c_ was a reliable measure of chronic hyperglycaemia, which is associated with long-term complications. HbA_1c_ was therefore suggested to be useable for diabetes diagnosis [18]. The expert committee also stated that those with HbA_1c_ levels from 42 mmol/l (6.0 %) to 47 mmol/mol (6.4%) should receive effective preventive interventions because of the relatively high likelihood of progressing to diabetes. They raised concerns regarding the use of the term ‘prediabetes’ to label this group of people, as not all people with HbA_1c_ in this range will develop diabetes. In 2010, the ADA widened the high-risk HbA_1c_ range to 5.7–6.4% (39–47 mmol/l) [19]. The EASD and IDF did not adopt these changes. When using HbA_1c_ (a proxy of blood glucose levels) as the primary diagnostic tool for (pre)diabetes, it is important to note that factors beyond the plasma glucose concentration contribute to the variation in HbA_1c_ [20], especially in the non-diabetic range [21]. Also, there are indications of a growing discrepancy between HbA_1c_ and other measures of blood glucose levels with increasing age, but this needs further investigation [22].

## The prevalence of prediabetes and the risk of future disease

On an individual level, blood glucose levels and the progression to type 2 diabetes are the results of a complex interplay between genetic makeup and the social and physical environment [23]. The prevalence of prediabetes, therefore, depends on the characteristics of the population as well as the diagnostic criteria used [7, 24]. Consequently, whether a given individual is labelled as having prediabetes rests in part on the cut-off points used in their country of residence, and it is often not possible to compare prevalence estimates across countries/studies. Yet, no matter the diagnostic criterion used, a substantial proportion of the world’s adult population has prediabetes defined as intermediate hyperglycaemia. For example, the estimated prevalence of prediabetes in adults has been reported to be ~50% in a large Chinese study and ~38% in the USA (both estimates are based on the ADA criteria for IFG, IGT and HbA_1c_-based prediabetes) [25, 26], and 17% in a Dutch cohort of adults aged 45–75 years (using the WHO IFG and IGT criteria) [27].

Depending on the definition used and the population examined, 10–50% of individuals with prediabetes will progress to overt diabetes within the next 5 to 10 years, with the highest risk in the group of individuals with combined IFG and IGT [28, 29]. Yet, even more individuals with prediabetes (around 30–60%) will revert to normoglycaemia within 1 to 5 years [28]. The high prevalence and the relatively low 5–10 year conversion rate to type 2 diabetes may, in part, be due to the cut-off points used to define prediabetes (particularly with the ADA/American Association of Clinical Endocrinologists criteria) falling within the reference limits for glucose levels reported in low-risk populations [30], especially with increasing age [31, 32]. The low cut-off points might also explain why 30% of young adults with a mean BMI of around 25 kg/m^2^, but with no other apparent risk factors for diabetes, have been reported to have ADA-defined prediabetic levels of HbA_1c_ or/and IFG in a population-based study from Liechtenstein [33]. Little is known about the lifetime risk of type 2 diabetes in people with prediabetes. In a Dutch study, the mean estimated lifetime risk of developing type 2 diabetes was highly dependent on body size. For those with IFG (based on the WHO criteria) and overweight/obesity (BMI >25 kg/m^2^), the risk was >75% at the age of 45 years, whereas it was ~36% in individuals with IFG and a BMI <25 kg/m^2^. Taking waist circumference into account further stratified the risk [34].

Prediabetes has been associated with a long list of current and future diseases, including cardiovascular disease, non-alcoholic fatty liver disease, neuropathy, chronic kidney disease, cancer and dementia, as well as all-cause mortality [5, 6, 27, 35]. The risk may be highest in those with IGT, though firm evidence is missing [5, 6]. As with diabetes, the development of these outcomes is the result of complex processes [23], and individuals with prediabetes represent a heterogenous group with varying risk of developing complications [35]. Results from Mendelian randomisation studies of variants that affect glycaemia indicate a causal relationship with coronary artery disease, even within the prediabetic range [36]. However, the associations reported in the literature are not strong enough to use prediabetes as a screening test for the risk of later complications [37].

## Intervening in people with prediabetes

Type 2 diabetes is preventable (or at least delayable) through intensive lifestyle changes in individuals with prediabetes, with the caveat that most trials have included people with IGT, often in combination with overweight [38]. Interventions aimed both at individuals at high risk of type 2 diabetes and at whole populations are reportedly cost-effective with regard to preventing diabetes [39], though some have questioned the translatability of the individual-level prevention trials to real-world conditions [10]. Programmes targeting single individuals fall into the category of high-risk prevention [40]. Due to the high prevalence of prediabetes, the potential target groups of these programmes include such a large proportion of the population that intervening in the entire target group would approach population-based prevention in scale, at least when using the ADA cut-off points. However, interventions targeting individuals do not have the benefits of population-based prevention, namely shifting the average risk of a population by targeting the underlying societal causes of disease development and, thereby, reducing the number of future cases [40]. In the well-known prevention trial, the Diabetes Prevention Program, the NNT to prevent one case of diabetes at the 3 year follow-up was 7, despite the participants going through a very substantial intervention programme [41]. The Diabetes Prevention Program included participants with slightly elevated fasting glucose levels, IGT and overweight, and the rate of conversion to diabetes was 29% in the control group [41]. A post hoc analysis from the trial indicated that those with the highest estimated risk benefitted the most [42]. Higher NNTs have been reported based on meta-analyses of trials applying lifestyle interventions in mixed settings [10, 43]. Barry et al reported an average NNT of 33 in trials lasting less than 3 years and an average NNT of 12 in trials lasting 3–6 years [10]. Most large prevention trials have included individuals with IGT [10] and it is questionable whether the results from trials recruiting people with IGT can be transferred to people with IFG or HbA_1c_-defined prediabetes [44]. Given the lower incidence of diabetes reported for people with HbA_1c_-based prediabetes and IFG [29], the NNT is expectedly higher in these groups [44].

The jury is still out when it comes to the ability of diabetes prevention programmes to prevent diabetes-related complications and other conditions associated with prediabetes [38]. We find it likely that such programmes can reduce the rate of these outcomes, but this will likely require lifelong support and, again, the NNT will be large if the target groups are people with prediabetes based on the current definitions.

## How to move on

Summing up, prediabetes is a major health burden associated with an increased risk/prevalence of subclinical metabolic disturbances and fulminant disease. However, the risk associated with prediabetes varies depending on the broader risk profile of the individual. Going forward, we believe the central challenge will be to strike a balance that reduces the health burden of prediabetes while also controlling costs and reducing medicalisation and unnecessary stigma.

**Fig. 1:**
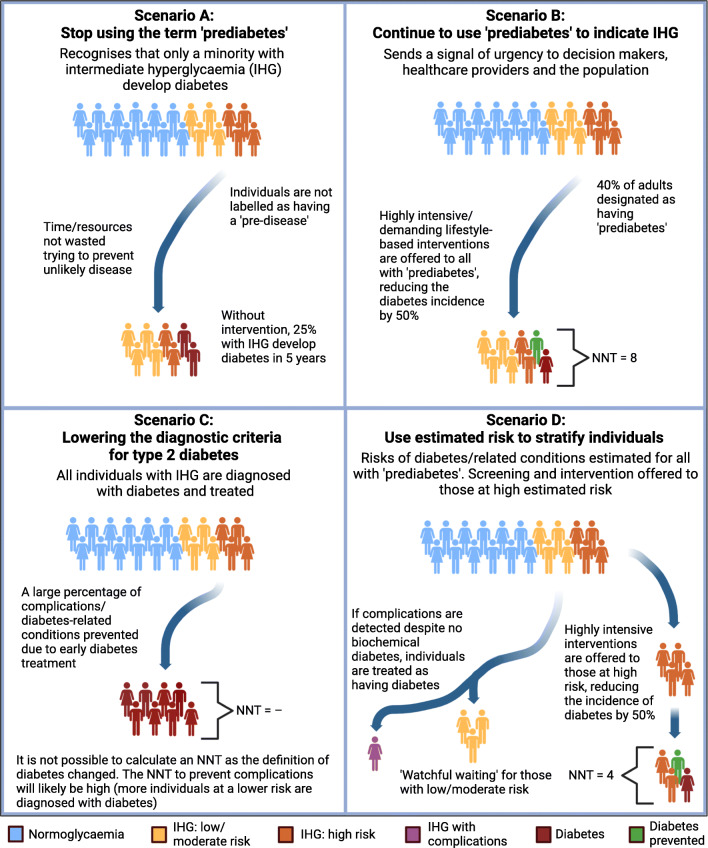
Illustration of the potential approaches to prediabetes. In a population with a prevalence of intermediate hyperglycaemia (IHG; ‘prediabetes’) of 40%, eight out 20 individuals have IHG. Assuming a diabetes incidence of 25% in those with IHG, 2/8 individuals with IHG (‘prediabetes’) will develop diabetes within 5 years if no individual-level intervention is offered (Scenario A). Offering lifestyle-based individual-level interventions will reduce the incidence of diabetes by 50%, but with a high NNT (NNT = 8/1 = 8; Scenario B). Lowering the diagnostic threshold of diabetes (Scenario C) has a large preventive potential but pathologises many. Stratifying preventive efforts based on estimated risk (Scenario D) has the potential to reduce the NNT. Those at high risk will be offered individual-level interventions, whereas those at low/moderate risk enter a surveillance programme (‘watchful waiting’). The higher the cut-off point used, the lower the NNT; however, more individuals that are not offered an intervention will develop diabetes. The estimated risk at which to offer individual-level intervention will need to strike a balance between the load on healthcare systems, preventing a substantial number of cases, and not leading to undertreatment. The illustration was created with BioRender.com

So how do we achieve this? Should we completely abandon the term prediabetes to indicate intermediate hyperglycaemia to avoid a label that seems to imply a pre-disease (Fig. 1 and ESM Table 1, scenario ‘A’)? Should we treat all people with prediabetes as being at high risk of diabetes and offer them individual-level interventions, as suggested, for instance, by the ADA [45] (Fig. 1 and ESM Table 1, scenario ‘B’)? Should we expand the diagnostic criteria of diabetes to include the prediabetic range, with a resulting large increase in the number of people with diabetes of whom many will have a low risk of developing complications (Fig. 1 and ESM Table 1, scenario ‘C’)? Or should we refine our approach to prediabetes by calculating the risk of developing diabetes, complications and related conditions, and only offer individual-level interventions to those at highest risk (Fig. 1 and ESM Table 1, scenario ‘D’). The financial and health impacts of the different scenarios presented in Fig. 1/ESM Table 1 will vary, but to what extent needs to be investigated. This will include an evaluation of how the financial and health impacts differ between low- and high-income countries and across different welfare/healthcare systems.

Prediabetes is indeed a controversial subject, and even among the authors of this article, there are diverging opinions. Some of us prefer to lower the diagnostic threshold of diabetes to include the prediabetic range (WHO cut-off points), whereas others prefer to reserve the term prediabetes to those of estimated high risk. However, no matter our personal standpoints, we cannot allow ourselves to ignore the substantial burden of disease caused by prediabetes/intermediate hyperglycaemia, both now and, even more so, in the future. In the face of the limited resources available and the severity of the matter, we suggest that the best compromise is to retain the term prediabetes in its current form, but adopt a stratified, precision medicine approach to screening and prevention based on estimated risk (as demonstrated by scenario ‘D’ in Fig. 1 and ESM Table 1). This recognises the varying risk among individuals with prediabetes and allows for the identification of those who develop complications, despite not having diabetes based on the biochemical definition.

The stratified approach will help ensure reasonable NNTs in preventive interventions while controlling costs. To realise this, we suggest moving towards stratifying those with prediabetes (and those without) based on short-term and lifetime estimated risk of diabetes, diabetes-related complications and other comorbidities. The estimated risk can serve as a basis for more informed individual-level conversations about the prevention of diabetes and related conditions, and thereby aid shared decision-making. We envision that most individuals at low to moderate risk will enter a form of ‘watchful waiting’ with a regular re-evaluation of their risk. Like with other risk factors for harder endpoints, such as hypertension and hyperlipidaemia, individuals with a high risk for diabetes and related complications will need lifelong support, including a focus on controlling body weight. Intervening before the development of overt diabetes will likely make the reversion to ‘normal’ glucose metabolism easier compared with attaining remission of diabetes.

As part of this approach, we suggest that individuals with a high estimated risk of prevalent diabetes-related complications are screened, and if complications exist, they must be treated as having overt diabetes despite having glycaemic levels below the diagnostic threshold for diabetes. Sticking to the biochemical diagnostic criteria in these cases is the same as ignoring that factors beyond blood glucose levels play a role in the development of diabetic complications. Studying the viability of a screen-and-treat approach for diabetic complications in high-risk individuals without biochemical diabetes will be an important avenue for future research. Our suggestion rests heavily on risk engines that can reliably identify the absolute risk of a series of outcomes, both future ones and those already established. This is partly possible using existing risk engines, such as QDiabetes [8] and similar models, but more advanced models are necessary to fully implement our suggestion. These risk engines must be able to robustly predict the risk of a range of relevant outcomes. The next step will be to establish the cut-offs for the estimated risk at which to offer individual-level interventions. This will require research into the cost–benefits, both for individuals with prediabetes (efforts vs potential gains in personal health) and for societies. We encourage the research community and decision makers to increase attention and resources towards this area. This should include a focus on how to improve the delivery and communication of the risk estimates to the individuals at risk. If implemented, the refinement of risk assessment will address some of the challenges arising from using the lower cut-off points for prediabetes suggested by the ADA, including the low/moderate positive predictive value for the development of diabetes.

The metabolic aberrations that lead to diabetes, diabetic complications and other related diseases are (likely) chronic in nature and need long-term, if not lifelong, interventions. At present, there is a lack of evidence for the long-term effectiveness of prevention programmes. Such trials are resource-demanding and require a long follow-up time. Stratification of treatment intensity by estimated risk should theoretically reduce the NNT and increase the likelihood that such interventions can reduce the incidence of adverse outcomes. However, this does not solve the problem of long-term adherence to treatments. Identifying interventions that are effective and tolerable over longer timespans in subgroups of people with prediabetes is an important avenue of research if we are to effectively reduce the health burden caused by prediabetes.

Importantly, the individual-level approach cannot stand alone, and we strongly encourage policymakers to prioritise population-based approaches. Population-level interventions have the potential to shift the risk distribution of a population and improve the average metabolic profile, which will benefit the general population [40]. Such interventions lie outside the realm of the healthcare system and rely on cross-sectorial collaborations with strong leadership from policymakers who must implement structural changes in society to promote healthier living. We, the medical/scientific community, are important advocates in this regard who can help guide policymakers in this direction.

In the meantime, we would like to remind all that even strong proponents of the use of the term prediabetes, such as the ADA, emphasise that prediabetes is not a condition, but a risk factor [45]. We, therefore, encourage all to show caution not to frame prediabetes as an actual pre-disease when addressing individuals, the public and decision makers.

In conclusion, we advocate for a more refined approach to risk and prevention in people with prediabetes to balance the resources spent by both individuals and societies.

## Supplementary information

ESM (PDF 106 kb)

## Abbreviations

FPG: Fasting plasma glucose
IFG: Impaired fasting glucose
IGT: Impaired glucose tolerance
NNT: Number needed-to-treat
