# Supplementary material for: The prediabetes conundrum: striking the balance between risk and resources
Source: Diabetologia. 2023 Mar 10;66(6):1016–23. doi: 10.1007/s00125-023-05890-y (PMC10163079; doi:10.1007/s00125-023-05890-y)
Supplement: Supplementary file 1 — (PDF 106 kb) [file 125_2023_5890_MOESM1_ESM.pdf]

Supplement

Table S1: Suggested approaches to prediabetes

Scenarios	A. Stop using the term prediabetes	B: Continue to use the term prediabetes	C: Lowering the diagnostic threshold for diabetes	D: Use estimated risk to stratify individuals
**Description**	Abandon the term prediabetes to recognize that only a minority of individuals with intermediate hyperglycaemia will develop diabetes.	Continue to use the term prediabetes to indicate intermediate hyperglycaemia.	Lower the diagnostic threshold for diabetes to include the prediabetic range.	Use the estimated risk of disease (e.g., diabetes-related complications) to stratify individuals with prediabetes.
**Pros**	• Individuals who will not develop diabetes are not labelled as having prediabetes and will not spend time & resources trying to prevent a disease they will not develop.<br>• Confusion about the different cut-points and definitions suggested by the professional societies will be avoided	• Using the term prediabetes sends a signal of urgency to decision-makers, healthcare providers and the population.<br>• Many who will develop diabetes/ complications will be identified (high sensitivity) and can be offered support to prevent disease.	• Many who will later develop complications will be identified (high sensitivity) and treated early.<br>• There is a potential for early treatment of risk factors associated with other non-communicable diseases.	• Acknowledging that many factors (e.g., age and BMI) influence disease development and progression.<br>• Reflects that risk is not dichotomous but continuous.<br>• Will likely improve the positive predictive value and hence reduce the NNT and medicalisation.
**Cons**	• Potentially less focus on preventing diabetes from society and politicians, because there is no longer a large group with a "pre-disease".<br>• Healthcare providers may have reduced focus on monitoring glycaemia and treating associated risk factors.	• Many who will not develop diabetes/ complications are labelled as having prediabetes (low specificity). This can lead to:<br>**a)** a low positive predictive value;<br>**b)** a substantial NNT to prevent one case of disease - time and resources wasted; and<br>**c)** potential negative psychological effects for many individuals (stigma).	• Many who will not develop complications are diagnosed with diabetes (low specificity). This can lead to:<br>**a)** medicalisation and stigmatisation;<br>**b)** increased cost for both society and the individual (cost of medication, lost income, higher insurance cost);<br>**c)** a substantial NNT to prevent one case of disease - time and resources wasted; and<br>**d)** potential side effects of unnecessary pharmacological treatment.	• Will require a reorientation from a dichotomous view of risk to a continuous one.<br>• Staff need to be educated in the use, interpretation, and communication of risk estimates.<br>• Users/patients need to be educated in using risk estimates in joint decision-making.<br>• It will add complexity and require more data to be fed to the risk model.
**Comments**		If this scenario is chosen, reaching consensus regarding the cut-points used and the interventions offered is essential. The cons are augmented when using the lower ADA endorsed		Such models need to be implemented in electronic patient record systems to be incorporated into a busy clinical workday.

		cut-points for fasting glucose and HbA1c.		
